# Non-Markovian equilibrium and non-equilibrium barrier-crossing kinetics in asymmetric double-well potentials

**DOI:** 10.1140/epje/s10189-025-00488-1

**Published:** 2025-05-22

**Authors:** Laura Lavacchi, Benjamin A. Dalton, Roland R. Netz

**Affiliations:** Fachbereich Physik, Freie Universität Berlin, Berlin, 14195 Germany

## Abstract

**Abstract:**

Barrier-crossing processes in nature are often non-Markovian and typically occur over an asymmetric double-well free-energy landscape. However, most theories and numerical studies on barrier-crossing rates assume symmetric free-energy profiles. Here, we use a one-dimensional generalized Langevin equation (GLE) to investigate non-Markovian reaction kinetics in asymmetric double-well potentials. We derive a general formula, confirmed by extensive simulations, that accurately predicts mean first-passage times from well to barrier top in an asymmetric double-well potential with arbitrary memory time and reaction coordinate mass. We extend our formalism to non-equilibrium non-Markovian systems, confirming its broad applicability to equilibrium and non-equilibrium systems in biology, chemistry, and physics.

**Graphic abstract:**

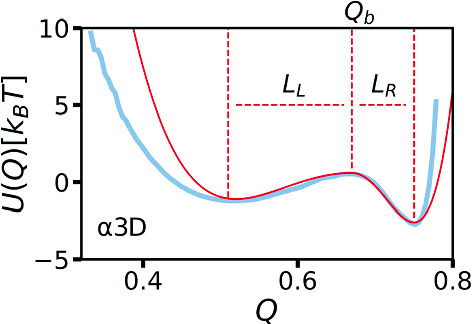

Introduction

The description of barrier-crossing phenomena has a long history in the physics and chemistry communities. Dating back to the works of Arrhenius [1], it has long been known that the barrier-crossing reaction rate scales as $\kappa \text {e}^{-\beta U_0}$, where $\beta =1/(k_\text {B}T)$ is the inverse thermal energy, $U_0$ is the height of the energy barrier characterizing the free-energy landscape, and $\kappa$ is a prefactor that depends not only on the free-energy profile, as in transition-state theory [2, 3], but also on the dissipative coupling to the environment. Kramers calculated the prefactor $\kappa$ using Markovian theory [4], where environmental dissipation is described by a time-independent friction coefficient $\gamma$, implying infinitely fast environmental relaxation dynamics. Kramers derived an explicit formula for the barrier-crossing rate in the high- and low-friction limits. The regime between these asymptotic limits, known as the Kramers turnover regime, was later described by Mel’nikov and Meshkov [5]. However, most environments do not display instantaneous relaxation. The influence of finite environmental relaxation times on reaction coordinate dynamics was described by Zwanzig [6] and Mori [7], who used projection techniques to show that the dynamics of a low-dimensional observable in a general many-body system is described by the generalized Langevin equation (GLE) with a time-dependent friction memory kernel $\Gamma (t)$, representing dynamic coupling to the dissipative environment [8]. Grote and Hynes (GH) derived a self-consistent equation for barrier-crossing rates in systems with short memory times in the medium- to high-friction regime [9], and Pollak, Grabert, and Hänggi subsequently constructed a theory suitable for arbitrary time-dependent memory kernels, also applicable for long memory times [10, 11].

**Fig. 1:**
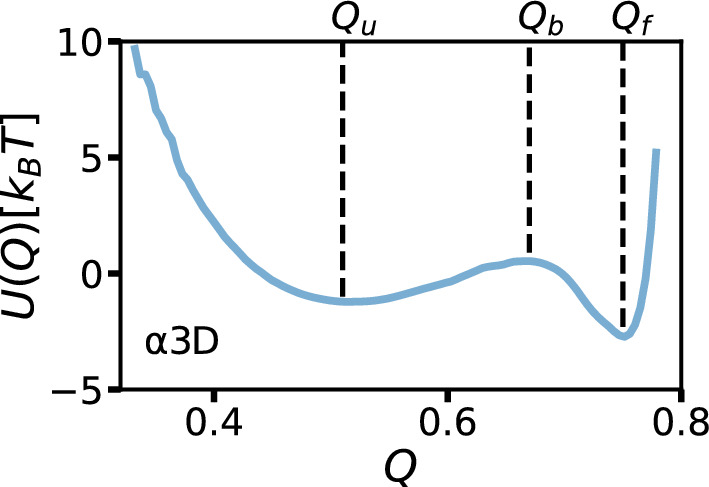
Free-energy profile for the fraction of native contacts reaction coordinate, *Q*, for the fast-folding de novo protein $\alpha _3$D, extracted from extensive molecular dynamics simulations originally published by Lindorff-Larsen et al. [20]. The free-energy profile for *Q*, taken from Dalton et al. [23], is given by $U(Q) = -k_\mathrm{{B}} T \text {log}[\rho (Q)]$, where $\rho (Q)$ is the probability density for the reaction coordinate. The unfolded $Q_u$, barrier top $Q_b$, and folded $Q_f$ reaction coordinate values are indicated. The free-energy profile parameters are the barrier heights $U_f = U(Q_b) - U(Q_f)$, $U_u = U(Q_b) - U(Q_u)$, and the barrier widths $L_f = Q_f - Q_b$, $L_u = Q_b - Q_u$. For $\alpha _3$D, $\beta U_f = 3.2$, $\beta U_u = 1.7$, $L_f = 0.08$, and $L_u = 0.15$ [23]

Many barrier-crossing rate theories represent the free-energy landscape as an inverted parabola. However, in many scenarios, relaxation in the potential wells is also important, and the free-energy landscape is more realistically modeled as a double-well potential, relevant, for example, to chemical reactions and protein folding [12–19]. Symmetric double-well free-energy profiles are typically assumed. In Fig. 1, we show the free-energy profile of the fast-folding de novo protein $\alpha _{3}$D, calculated from all-atom MD simulation trajectories in an explicit-water solvent, originally published by Lindorff-Larsen et al. [20]. The free-energy profile is calculated along the one-dimensional reaction coordinate, the fraction of native contacts, *Q* [21, 22], according to $U(Q)=-k_BT\text {log}[\rho (Q)]$, where $\rho (Q)$ is the probability density for the *Q* reaction coordinate [23]. $\alpha _3$D is a two-state folder, exhibiting distinct folded and unfolded states with a pronounced separating barrier, such that the protein folds and unfolds by traversing the energy barrier between the two states. While the barrier region can be well represented as an inverted parabola, the free-energy landscape, including the wells, is strongly asymmetric. In this article, we investigate non-Markovian barrier-crossing dynamics in asymmetric double-well potentials, as presented in Fig. 1 [24, 25]. Using extensive non-Markovian simulations, we parameterize an asymptotic formula describing the time required to reach the barrier top from a potential well in the presence of exponentially decaying memory. This formula is based on the observation that even in strongly non-Markovian systems, the dynamics in the two wells of an asymmetric double-well potential are decoupled, meaning the shape of one potential well does not influence the time needed to reach the barrier top starting from the other well. In the final section, we investigate barrier-crossing kinetics in asymmetric double-well potentials for non-equilibrium systems.

## Model

As shown previously, protein folding, molecular conformational transitions, such as dihedral isomerization, and many other dynamic processes are strongly non-Markovian processes [23, 26–31]. In fact, the dynamics of fast-folding proteins are well described by a theory that explicitly accounts for multi-exponential memory [23, 28, 30]. To model memory-dependent friction effects on barrier-crossing dynamics in an asymmetric double-well potential, we simulate the generalized Langevin equation (GLE) in the linear-friction approximation [6, 7, 32, 33] in the potential *U*(*x*),

1 $$\begin{aligned} m\ddot{x}(t) = - \int _{0}^t \Gamma (t-t^\prime ) \dot{x}(t^\prime ) dt^\prime -\nabla U\big (x(t)\big )+ F_R(t), \end{aligned}$$

where *m* is the effective mass, assumed not to depend on the reaction coordinate, $\Gamma (t)$ is the friction memory kernel, and $F_R(t)$ denotes the random force, which approximately satisfies the relation [34, 35]

2 $$\begin{aligned} \langle F_R(t)F_R(t^\prime )\rangle = \beta ^{-1}\Gamma (t-t^\prime ), \end{aligned}$$

with $\beta = 1/k_B T$ the inverse thermal energy. For simplicity, in the simulations we choose a single-exponential memory kernel

**Fig. 2:**
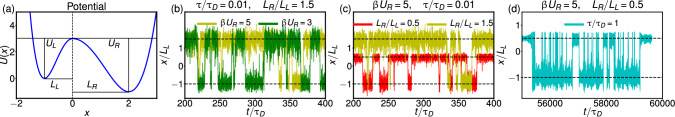
**(a)** The asymmetric double-well potential defined in Eq. (4) depends on four parameters: the distances from the left and right minima to the barrier top, $L_L$ and $L_R$, respectively, and the barrier heights in the left and right wells, $U_L$ and $U_R$. Trajectories for systems with different parameters are shown in **(b)**, **(c)**, and **(d)**. Identical colors represent trajectories with identical parameters. For all trajectories, we choose $\beta U_L = 3$ and $\tau _m/\tau _D = 0.01$. In **(b)**, the memory time is set to $\tau /\tau _D = 0.01$ and $L_R/L_L = 1.5$, for $\beta U_R = 3$ and $\beta U_R = 5$. In **(c)**, $\beta U_R = 5$ and $\tau /\tau _D = 0.01$, for $L_R/L_L = 0.5$ and $L_R/L_L = 1.5$. In **(d)**, $\beta U_R = 5$, $L_R/L_L = 0.5$ for a long memory time $\tau /\tau _D = 1$

3 $$\begin{aligned} \Gamma (t) = \frac{\gamma }{\tau } e^{-\frac{|t|}{\tau }}, \end{aligned}$$

where $\tau$ is the memory time and $\gamma = \int _0^{\infty }dt\Gamma (t)$ is the friction coefficient. For the potential *U*(*x*), we use the specific asymmetric double-well potential

4 $$\begin{aligned} U(x) = {\left\{ \begin{array}{ll} U_L \left[ \left( \frac{x}{L_L}\right) ^2-1\right] ^2 &  \text{ if } x \le 0 \\ U_R \left[ \left( \frac{x}{L_R}\right) ^2-1\right] ^2 + (U_L-U_R)&  \text{ if } x>0, \end{array}\right. } \end{aligned}$$

where $L_L$ and $L_R$ are the distances from the left and right minima to the barrier top, characterizing the well widths, respectively, and $U_L$ and $U_R$ are the barrier heights viewed from the left and right wells, respectively, see Fig. 2(a). In Appendix A, we demonstrate that the free-energy profile of the $\alpha _{3}$D protein, shown in Fig. 1, is well approximated by Eq. (4). The potential is designed such that the potential itself and its first derivative are continuous, while its second derivative is discontinuous at the barrier top, which, however, does not perturb the reaction coordinate dynamics. We note that our results pertain to this specific choice of potential but should become universally valid for large barrier heights, since in this limit barrier-crossing dynamics are expected to only depend on the barrier heights $U_L$ and $U_R$ and the effective well widths $L_L$ and $L_R$ [36]. Two additional characteristic timescales are the inertial time $\tau _m = m/\gamma$ and the diffusion time $\tau _D = \beta L_L^2 \gamma$, where the width of the left well $L_L$ is chosen as the characteristic length scale.

In Fig. 2, we show trajectories for different memory times, barrier heights, and potential widths to illustrate the influence of the parameters $\tau$, $U_L$, $U_R$, $L_L$, and $L_R$ on the dynamics of the reaction coordinate *x*(*t*). In Fig. 2(b), we fix the memory time $\tau$ and the potential widths $L_L$ and $L_R$ and vary the barrier heights. We observe that for increased right barrier height, with $\beta U_R = 5$ and $\beta U_L = 3$, the system spends more time in the right well than in the left well; in the symmetric case, $\beta U_R = 3$ and $\beta U_L = 3$, the time spent in each well is the same. In Fig. 2(c), we vary the ratio of the left and right potential widths. In Fig. 2(d), we show a trajectory for a long memory time, $\tau /\tau _D = 1.0$, and all other parameters the same as the red curve in Fig. 2(c). We observe frequent state recrossings, reminiscent of inertial dynamics, which are typical for strongly non-Markovian systems [37]. Overall, Fig. 2(b)-(d) illustrates how barrier heights, potential widths, and memory time affect non-Markovian system dynamics in asymmetric double-well potentials, demonstrating that all of these parameters influence the barrier-crossing dynamics.

## Results

### Well-to-well and well-to-barrier-top transition times for symmetric double-well potentials

We first treat symmetric potentials, for which $U_L=U_R=U_0$ and $L_L=L_R$, and compare well-to-well and well-to-barrier-top transition times. Recently, asymptotic formulas for predicting transition times in non-Markovian systems, accounting for particle mass, amplitude, and the memory time of the friction kernel in symmetric double-well potentials, have been derived [38–41]. These formulas are constructed from the high- and low-friction limits of the Markovian Kramers prediction for the well-to-well mean first-passage time (MFPT) [4], which can be combined into an interpolating crossover formula according to

5 $$\begin{aligned} \tau _{MFP} = e^{\beta U_0} \left( d_1 \frac{m_{\text {eff}}^L}{\beta U_0 \gamma _{\text {eff}}^{L}} + d_2 \frac{\gamma _{\text {eff}}^{H}}{K} + 4 \sqrt{2\frac{m}{K}}\right) . \end{aligned}$$

Here, the first term corresponds to the low-friction inertial limit, the second term to the high-friction overdamped limit, and the third heuristic crossover term accounts for the Kramers turnover regime. According to Kramers’ theory [4], the constants $d_1$ and $d_2$ are given by $d_1 = 3\pi /(8\sqrt{2})$ and $d_2 = 2 \sqrt{2} \pi$, where $K = U^{\prime \prime }(L)= 8 U_0/L^2$ is the curvature of the potential *U*(*x*) at the well minimum. For a Markovian system, the effective mass $m_{\text {eff}}$ and effective friction $\gamma _{\text {eff}}$ are given by their bare values. For general non-Markovian systems, $m_{\text {eff}}$ and $\gamma _{\text {eff}}$ account for non-Markovian effects and are determined from the two-point positional autocorrelation functions derived from the GLE in Eq. (1), as discussed in detail in Appendix B (see also [38–41]). By inserting the low-friction effective expressions $\gamma _\text {eff}^L$ and $m_\text {eff}^L$ into the inertial contribution and the high-friction expression $\gamma _\text {eff}^H$ into the high-friction contribution in Eq. (5), we can rearrange and fit the numerical constants to simulation data to achieve

6 $$\begin{aligned} \tau _{MFP}= &   e^{\beta U_0} \left[ \frac{1}{\beta U_0} \frac{3 \pi }{8\sqrt{2}} \left( \frac{m}{\gamma } + \frac{2K \tau ^2}{3\gamma }\right) \right. \nonumber \\  &   \quad +\left. \frac{2\sqrt{2}\pi \gamma }{K}\frac{1}{1+ K\beta U_0 \tau /(4\gamma )} + 4 \sqrt{2\frac{ m}{K}}\right] .\nonumber \\ \end{aligned}$$

When comparing with the formulas proposed by Lavacchi et al. [41] and by Kappler et al. [39], the most significant difference is in the term that describes the memory-induced speed-up, i.e., the $\tau$-dependent term in the denominator of the second term in Eq. (6). While in [39], this term is linear in the barrier height $U_0$, and in [41], it is independent of $U_0$, in Eq. (6), this term exhibits a quadratic dependence on $U_0$ (keeping in mind that *K* is proportional to $U_0$). These differences have the most pronounced effect for memory-induced speed-up effects in the small mass regime, which in fact is the most relevant regime for protein folding [23]. In Appendix C, we compare Eq. (6) and the formulas presented in [39] and [41] with simulation data, showing that Eq. (6) is most accurate for predicting MFPTs in symmetric double-well potentials across a range of memory times, particle masses, and barrier heights.

**Fig. 3:**
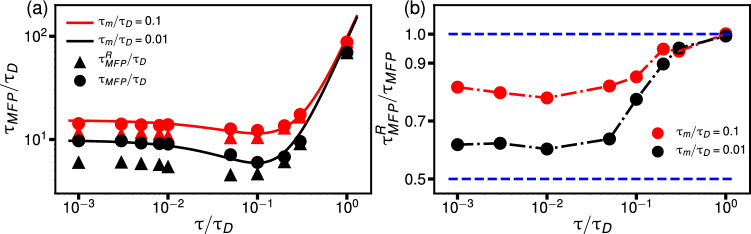
Comparison between well-to-well and well-to-barrier-top MFPTs for a symmetric double-well potential with barrier height $\beta U_0 = 3$. **(a)** Well-to-well MFPTs from simulations ($\tau _{MFP}$, circles), plotted as a function of the rescaled memory time $\tau /\tau _D$, agree well with the predictions of Eq. (6) (solid lines). Triangles represent simulation results for the well-to-barrier-top MFPTs $\tau ^R_{MFP}$. The results are shown for two rescaled masses. **(b)** Simulation results for $\tau _{MFP}^R/\tau _{MFP}$ as a function of rescaled memory time, with dashed-dotted lines as guides to the eye. The horizontal dashed lines indicate the limits $\tau _{MFP}^R/\tau _{MFP} \rightarrow 1$ as $\tau /\tau _D \rightarrow \infty$ and $\tau _{MFP}^R/\tau _{MFP} = 0.5$, expected when $\tau _m/\tau _D \rightarrow 0$ and $\tau /\tau _D \rightarrow 0$ for $\beta U_0 \gg 1$

In Fig. 3(a), we show well-to-well MFPTs for simulations of Eq. (1) in a symmetric double well as a function of the rescaled memory time $\tau /\tau _D$ for a fixed barrier height $\beta U_L = 3$ and two different inertial times. For the calculation of $\tau _{MFP}$, we take the mean of all passage times between crossing the minimum of one free-energy and reaching the minimum of the other well for the first time, which is the so-called mean all-to-first-passage time [37]. The prediction by Eq. (6) agrees well with the simulation results for both $\tau _m$, accurately capturing the memory-induced speed-up regime for intermediate memory time. In Appendix D, we compare the simulation results to GH theory, which is known to break down for long memory times [38, 42]. There, we explore the limits of GH theory and discuss deviations from simulation results for intermediate and long memory times. We also calculate MFPTs for transitions from a well minimum to the barrier top, denoted as $\tau _{MFP}^L$ and $\tau _{MFP}^R$ for transitions from the left and right wells, respectively, referred to as well-to-barrier-top transition times. Distinguishing $\tau _{MFP}^L$ and $\tau _{MFP}^R$ for asymmetric potentials allows us to describe the dynamics in each well separately. For symmetric potentials and overdamped Markovian systems, i.e., in the double limit $\tau /\tau _D \rightarrow 0$ and $\tau _m/\tau _D \rightarrow 0$, the well-to-barrier-top transition time is exactly half of the well-to-well transition time for sufficiently high barriers [4, 43]

7 $$\begin{aligned} \tau _{MFP}^L= \tau _{MFP}^R = \tau _{MFP}/2. \end{aligned}$$

We test this relation for general non-Markovian systems in Fig. 3(a). The red and black lines show predictions from Eq. (6); the filled circles represent simulation data for the well-to-well MFPT, $\tau _{MFP}$, and the triangles represent the well-to-barrier-top MFPT, $\tau _{MFP}^R$. (We exclude $\tau _{MFP}^L$ because for a symmetric *U*(*x*), $\tau _{MFP}^R = \tau _{MFP}^L$.) In the short memory time limit, there is a pronounced difference between $\tau _{MFP}$ and $\tau _{MFP}^R$, particularly for the system with smaller mass. These differences are clearer in Fig. 3(b), which shows the ratio $\tau _{MFP}^R / \tau _{MFP}$ for two values of $\tau _m/\tau _D$ as a function of the rescaled memory time $\tau /\tau _D$. As the mass decreases from $\tau _m/\tau _D = 0.1$ to $\tau _m/\tau _D = 0.01$, we indeed see that $\tau _{MFP}^R/\tau _{MFP}$ approaches 0.5 for small memory times. The noticeable deviations from Eq. (7) even for $\tau _m/\tau _D = 0.01$ are due to finite-mass effects and to the fact that the potential barrier is not infinitely high. Reaching the overdamped high-barrier limit in simulations is difficult since small mass and high barriers require very long simulation times in order to equilibrate properly. $\tau _{MFP}^R/\tau _{MFP}$ remains approximately constant for small memory times but increases sharply around $\tau /\tau _D = 0.1$. For larger memory times, $\tau _{MFP}^R/\tau _{MFP}$ approaches unity for both values of $\tau _m/\tau _D$. This result shows that we cannot simply combine Eq. (7) with the formula for the well-to-well MFPT Eq. (6) to predict well-to-barrier-top transition times. Instead, we must construct distinct formulas for both $\tau _{MFP}^R$ and $\tau _{MFP}^L$ that are suitable for general $\tau$ and $\tau _m$ in asymmetric double-well potentials.

###  Well-to-barrier-top transition times for asymmetric double-well potentials

In Fig. 3(b), we demonstrate that for short memory times, $\tau _{MFP}^L$ and $\tau _{MFP}^R$ deviate from $\tau _{MFP}$ and are expected to approach $\tau _{MFP}^{L} = \tau _{MFP}^{R} = \tau _{MFP}/2$ in the zero-mass or high-friction limit for high enough barriers.

Therefore, we add a factor of 1/2 to the high friction term proportional to $\gamma /K$ in Eq.(6) but do not modify the high-mass term. To account for barrier asymmetry, i.e., differing barrier heights and potential widths between the two wells, we introduce left- and right-specific constants, $U_{L,R}$ and $K_{L,R} = 8U_{L,R}/L^2_{L,R}$. This modification to Eq. (6) yields a formula for the MFPTs of well-to-barrier-top transitions in the left and right wells of an asymmetric double-well potential

**Fig. 4:**
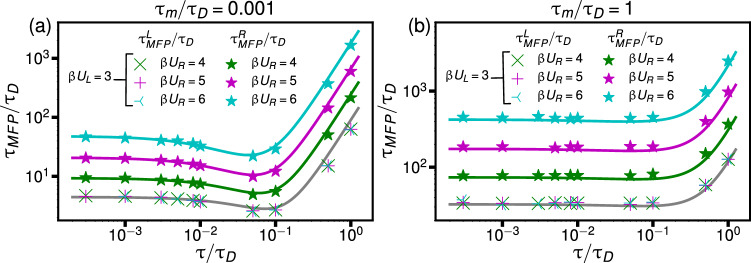
Well-to-barrier-top MFPTs as a function of $\tau /\tau _D$ for asymmetric double-well potentials with equal potential widths $L_L = L_R$. The left well has a fixed barrier height of $\beta U_L = 3$. Results are shown for different barrier heights of the right well: $\beta U_R = 4$ (green), $\beta U_R = 5$ (magenta), and $\beta U_R = 6$ (light blue). The MFPTs $\tau _{MFP}^R/\tau _D$ from simulations (colored symbols) are compared to predictions of Eq. (8) (colored lines). The gray lines show $\tau _{MFP}^L/\tau _D$ given by Eq. (8), which are independent of $\beta U_R$. Simulation results for $\tau _{MFP}^L/\tau _D$ are shown as colored symbols and are found to overlap, confirming the insensitivity of $\tau _{MFP}^L/\tau _D$ to $\beta U_R$. Results for two different masses are displayed in **(a)** for $\tau _m/\tau _D = 0.001$ and in **(b)** for $\tau _m/\tau _D = 1$

8 $$\begin{aligned} \tau _{MFP}^{L,R}= &   e^{\beta U_{L,R}} \left[ \frac{1}{\beta U_{L,R}} \frac{3 \pi }{8\sqrt{2}} \left( \frac{m}{\gamma } + \frac{2K_{L,R} \tau ^2}{3\gamma }\right) \right. \nonumber \\  &   \quad +\left. \frac{\sqrt{2}\pi \gamma }{K_{L,R}}\frac{1}{1\!+\! \beta U_{L,R} K_{L,R}\!\tau /(4\gamma )} \right. \nonumber \\  &   \quad \left. + 4 \sqrt{2\frac{ m}{K_{L,R}}}\right] . \end{aligned}$$

**Fig. 5:**
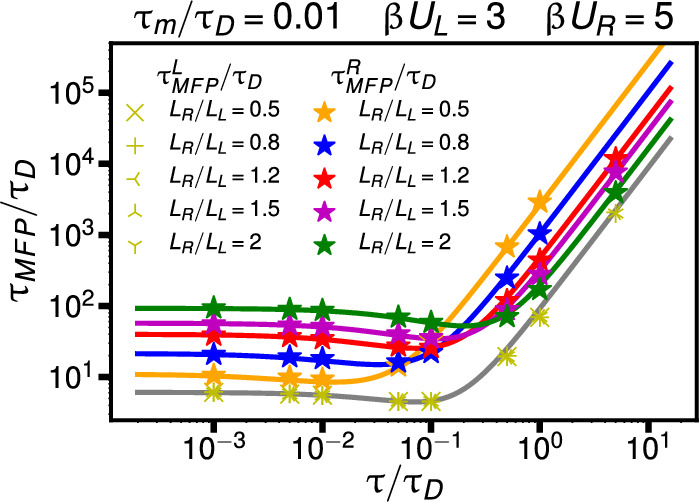
The well-to-barrier-top MFPT as a function of the rescaled memory time $\tau /\tau _D$ in asymmetric double-well potentials with varying potential width ratios $L_R/L_L$. The rescaled mass is set to $\tau _m/\tau _D = 0.01$, and the barrier heights are set to $\beta U_L = 3$ and $\beta U_R = 5$. Colored lines show predictions of Eq. (8), while colored symbols denote simulation results

In Fig. 4, we compare Eq. (8) to simulation results as a function of the rescaled memory time $\tau /\tau _D$ for fixed $L_L = L_R$, $\beta U_L = 3$, and varying $\beta U_R$. We observe excellent agreement for $\tau _m/\tau _D = 0.001$ in Fig. 4(a) and for $\tau _m/\tau _D = 1.0$ in Fig. 4(b), for both $\tau _{MFP}^{L}$ and $\tau _{MFP}^{R}$. In Fig. 5, we show $\tau _{MFP}^{R}/\tau _D$ and $\tau _{MFP}^{L}/\tau _D$ as a function of the rescaled memory time $\tau /\tau _D$ for fixed $\beta U_L = 3$, $\beta U_R = 5$, $\tau _m/\tau _D = 0.01$, and varying $L_R/L_L$. Asymmetric double-well potentials with unequal potential widths $L_L \ne L_R$ are relevant for protein folding, as demonstrated in Fig. 1. We see that the predictions of Eq. (8) for $\tau _{MFP}^{R}/\tau _D$ (colored lines) are in excellent agreement with the simulation results (symbols). The prediction $\tau _{MFP}^L/\tau _D$ from Eq. (8) (gray line) is independent of $L_R/L_L$ which is confirmed by the simulations, which demonstrate that results for $\tau _{MFP}^{L}/\tau _D$ overlap for all values of $L_R/L_L$. Thus, the MFPT to reach the barrier top from the left well $\tau _{MFP}^{L}$ is completely insensitive to the potential shape of the right well, quantified by $\beta U_R$ and $L_R$, from which we conclude that the barrier-crossing dynamics in one well is independent of the potential parameters in the other well, even for highly non-Markovian and highly inertial systems. This decoupling of the barrier-crossing kinetics in the two wells motivates deriving the asymptotic expression for the well-to-barrier-top MFPT in Eq. (8) and explains why this expression is useful for describing transition kinetics in asymmetric potentials.

Following previous arguments [39, 40], we generalize Eq. (8) to multi-exponential memory with multiple memory time scales. In this case, the memory kernel takes the form

9 $$\begin{aligned} \Gamma (t) = \sum _{i=1}^N \frac{\gamma _i}{\tau _i}e^{-\frac{|t|}{\tau _i}}, \end{aligned}$$

where $\gamma = \sum \gamma _i$, and the random force is given by $F_R(t) = \sum f_{R_i}(t)$ with components that satisfy

10 $$\begin{aligned} \langle f_{R_i}(t)f_{R_j}(t^{\prime }) \rangle = k_BT\frac{\gamma _i}{\tau _i}e^{-|t-t^{\prime }|/(\tau _i)}\delta _{i,j}. \end{aligned}$$

To obtain a formula for the MFPT to the barrier top for multi-exponential memory kernel, we decompose Eq. (8) into two contributions, the overdamped contribution

11 $$\begin{aligned} \tau ^{L,R}_{OD_i} =e^{\beta U_{L,R}}\! \Bigg [ \frac{\sqrt{2}\pi \gamma _i}{K_{L,R}} \frac{1}{1\!+\! \frac{\beta U_{L,R} K_{L,R}\tau _i}{4\gamma _i}} \!+\! 2 \sqrt{2\frac{ m}{K_{L,R}}}\! \Bigg ],\nonumber \\ \end{aligned}$$

and the energy-diffusion contribution

12 $$\begin{aligned} \tau ^{L,R}_{ED_i} =e^{\beta U_{L,R}}\! \Bigg [ \frac{1}{\beta U_{L,R}} \frac{3 \pi }{8\sqrt{2}} \left( \frac{m}{\gamma _i} \!+ \!\frac{2K_{L,R} \tau _i^2}{3\gamma _i}\right) \!+\! 2 \sqrt{2\frac{ m}{K_{L,R}}}\! \Bigg ].\nonumber \\ \end{aligned}$$

The well-to-barrier-top MFPT for a multi-exponential kernel is then given by adding the sum of the overdamped contributions and the inverse of the sum of the reciprocals of the energy-diffusion contributions according to [39, 40]

13 $$\begin{aligned} \tau ^{L,R}_{MFP} = \sum _{i=1}^N \tau ^{L,R}_{OD_i} +\left( \sum _{i=1}^N 1/ \tau ^{L,R}_{ED_i} \right) ^{-1}. \end{aligned}$$

Obviously, for a single-component exponential memory with $N=1$, we recover the original formula Eq. (8).

### Non-equilibrium transition times

So far, we have considered reaction times of equilibrium non-Markovian systems. Non-equilibrium systems are pervasive in biology, with numerous examples of non-equilibrium reaction kinetics [44–51]. Following recent results for the barrier-crossing dynamics of non-equilibrium systems in symmetric double-well potentials [41], we extend our current analysis of transition times in asymmetric potentials to non-equilibrium systems. We consider the GLE

14 $$\begin{aligned} m\ddot{x}(t) = - \int _{0}^t \Gamma _V(t-t') \dot{x}(t') dt' -\nabla U(x(t))+ F_R(t) \end{aligned}$$

with a random-force autocorrelation function given by

15 $$\begin{aligned} \langle F_R(t)F_R(t')\rangle = \beta ^{-1}\Gamma _R(t-t'), \end{aligned}$$

which does not necessarily satisfy the relation Eq. (2) but is characterized more generally by $\Gamma _V(t) \ne \Gamma _R(t)$. We note that the equality $\Gamma _V(t) = \Gamma _R(t)$ also breaks down for more complicated GLEs that involve a position-dependent mass or a nonlinear friction kernel even for equilibrium systems [34, 35, 52] and that the GLE derived by projection from a time-dependent Hamiltonian, which can be viewed as the first-principle description of a non-equilibrium system, is much more complicated than the GLE in Eq. (14) [53]. Nevertheless, Eqs. (14) and (15) for $\Gamma _V(t) \ne \Gamma _R(t)$ have been established as a standard heuristic model to describe non-equilibrium systems [48, 54–57]. Indeed, for $\Gamma _V(t) \ne \Gamma _R(t)$ the distribution of the reaction coordinate following from the GLE in Eq. (14) will not be given by the equilibrium Boltzmann distribution $\rho _{eq} (x) \sim e^{-\beta U(x)}$, which is the signature of a non-equilibrium system [58]. For simplicity, we assume the friction kernel and the random-force autocorrelation function to be given by exponential functions

16 $$\begin{aligned} \begin{aligned} \Gamma _V(t)&= \frac{\gamma }{\tau _V} e^{-\frac{|t|}{\tau _V}},\\ \Gamma _R(t)&= \frac{\gamma }{\tau _R} e^{-\frac{|t|}{\tau _R}}. \end{aligned} \end{aligned}$$

In equilibrium, $\tau _V = \tau _R$, while for a decay time of the random-force autocorrelation function $\tau _R$ differing from the decay time of the memory kernel $\tau _V$, we have a non-equilibrium system. Note that we choose the integrals over the friction kernel and the random-force autocorrelation function to be equal to $\gamma$. This does not restrict the generality of our model, since the factor $\beta$ in Eq. (15) (which represents the inverse thermal energy for equilibrium systems) can be chosen arbitrarily. As shown previously [41] and derived in Appendix E, for the specific non-equilibrium model defined by exponential kernels as given in Eq. (16), the inverse temperature $\beta$ in Eq. (8) is replaced by $\beta _{NEQ} =\beta \tau _R^2/\tau _V^2$ and the well-to-barrier-top mean first passage for the non-equilibrium system is in harmonic approximation given by

**Fig. 6:**
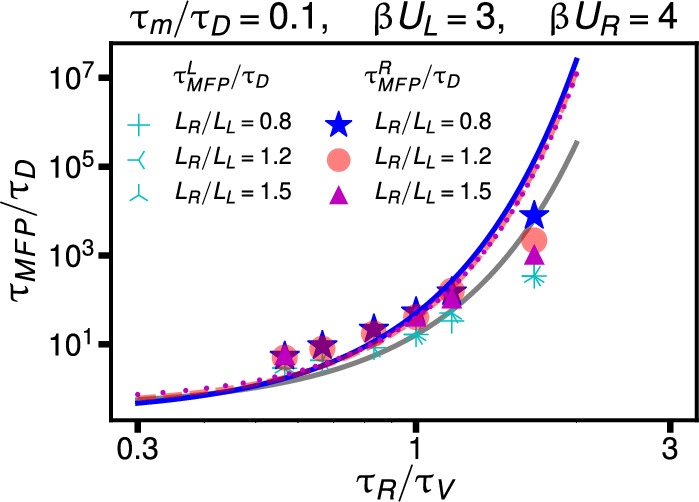
Non-equilibrium well-to-barrier-top MFPT as a function of the timescale ratio $\tau _R/\tau _V$ for various potential width ratios $L_R/L_L$. The barrier heights are $\beta U_L = 3$ and $\beta U_R = 4$, and the rescaled mass is $\tau _m/\tau _D = 0.1$. Symbols denote simulation results as indicated in the legend. The gray line depicts $\tau _{MFP}^L/\tau _D$, and the other colored lines show $\tau _{MFP}^R/\tau _D$ predicted by Eq. (17) for the various potential width ratios

$$\begin{aligned}  &   \tau _{MFP}^{L,R} = e^{\beta _{NEQ} U_{L,R}} \left[ \frac{1}{\beta _{NEQ} U_{L,R}} \frac{3 \pi }{8\sqrt{2}} \right. \\  &   \qquad \left. \left( \frac{m}{\gamma } + \frac{2K_{L,R} \tau _V^2}{3\gamma }\right) \right. \\  &   \quad \quad + \left. \frac{\sqrt{2}\pi \gamma }{K_{L,R}}\frac{1}{1+\beta _{NEQ} U_{L,R} K_{L,R}\tau _V /(4\gamma )} \right. \\  &   \qquad \left. + 4 \sqrt{2\frac{ m}{K_{L,R}}}\right] . \end{aligned}$$

In Fig. 6, we show $\tau _{MFP}^{R}/\tau _D$ and $\tau _{MFP}^{L}/\tau _D$ as a function of $\tau _R/\tau _V$ for fixed $U_R$, $U_L$, and $L_L$, for different values of $L_R/L_L$, noting that equilibrium is recovered for $\tau _R/\tau _V=1$. We see that $\tau _{MFP}$ increases as $\tau _R/\tau _V$ increases, and that the simulation data agree well with Eq. (17) for $\tau _R/\tau _V$ not too different from unity. The dependence of the transition times on the non-equilibrium effective temperature $\beta _{NEQ}=\beta \tau _R^2/\tau _V^2$ dominates in Eq. (17), and the dependence on the potential width ratio $L_R/L_L$ is rather minor. Therefore, the three lines in Fig. 6 are rather similar. In conclusion, Eq. (17) describes the transition time for a non-equilibrium system in an asymmetric double-well potential rather well; the main effect of non-equilibrium is to introduce an effective temperature that is proportional to the square of the ratio of the exponential memory-friction and random-force decay times, which is a result specific to our non-equilibrium exponential kernel model.

## Conclusion

We investigate non-Markovian reaction kinetics in asymmetric double-well potentials, based on the generalized Langevin equation (GLE), and we explore how asymmetries in barrier height and potential width, combined with memory effects, influence barrier-crossing dynamics. We first compare well-to-well transition MFPTs and well-to-barrier-top transition MFPTs, and we demonstrate that only for overdamped Markovian systems with high barriers does the well-to-barrier-top transition time approximate half of the well-to-well transition time. We show that the reaction dynamics in one well are largely independent of the other well’s parameters, allowing us to describe the well-to-barrier-top MFPT for each well separately. Based on this insight, we derive a general formula for the well-to-barrier-top MFPT using a harmonic approximation for the two-point correlation function, which proves to be accurate when compared with extensive GLE simulations. Our expression for the MFPT is derived specifically for the potential defined in Eq. (4), but in the high-barrier limit is expected to hold for general potentials. We extend our analysis to non-equilibrium systems, where the harmonic analysis of the non-equilibrium GLE yields a closed-form MFPT expression featuring an effective temperature, which we also validate by comparing with extensive GLE simulations. In summary, our work provides a robust framework for predicting reaction times in non-Markovian systems with asymmetric potentials, relevant across a range of biological, chemical, and physical systems, offering a more comprehensive understanding of reaction kinetics in complex environments.

## Figures and Tables

**Fig. 7:**
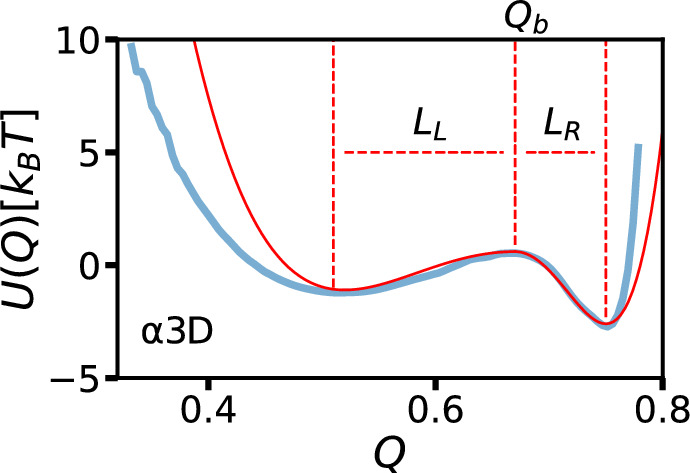
Free-energy profile for the fraction of native contacts reaction coordinate, *Q*, for the fast-folding $\alpha _3$D protein. The free-energy profile for *Q* is taken from [23] (blue line, as in Fig. 1). The original all-atom simulation data are from [20]. The red line shows the fitted asymmetric double-well potential according to Eq. (A.1)

**Fig. 8:**
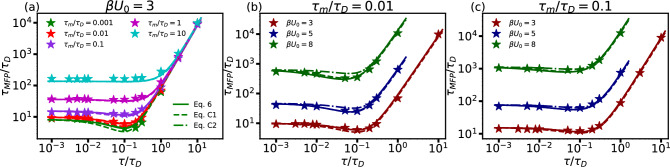
**(a)** Well-to-well MFPT as a function of $\tau /\tau _D$ for a symmetric double-well potential with $\beta U_0 = 3$ for various values of $\tau _m/\tau _D$. Comparison of simulation results (stars), Eq. (6) (continuous lines), Eq. (C.1) (dashed lines) and Eq. (C.2) (dashed-dotted lines). In **(b)** and **(c)**, MFPTs are shown for $\tau _m/\tau _D = 0.01$ and $\tau _m/\tau _D = 0.1$, respectively, for three different barrier heights

**Fig. 9:**
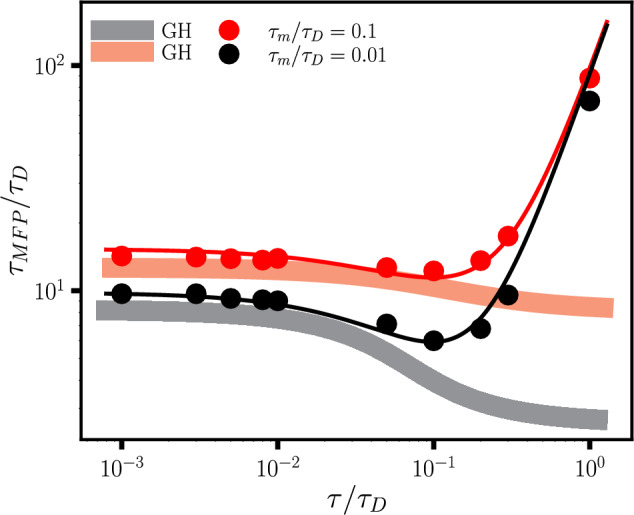
Comparison of well-to-well MFPTs for a symmetric double-well potential with barrier height $\beta U_0 = 3$ as a function of the rescaled memory time $\tau /\tau _D$ from simulations (circles) with predictions of Eq. (6) (thin solid lines) and with GH prediction of Eq. (D1) (thick lines). Results are presented for two different rescaled masses

## Fit of asymmetric potential to protein simulation data

In Fig. 7, we show by the blue line the free-energy profile for the fraction of native contacts reaction coordinate *Q*, evaluated for the $\alpha _3$D protein, which is the same data as shown in Fig. 1. The red line is the asymmetrically parameterized double-well fit according to

A.1 $$\begin{aligned} U(Q) = {\left\{ \begin{array}{ll} U_L\left[ \left( \frac{Q-Q_b}{L_L}\right) ^2-1\right] ^2 &  \text{ if } Q<Q_b \\ U_R \left[ \left( \frac{Q-Q_b}{L_R}\right) ^2-1\right] ^2 +(U_L -U_R) &  \text{ if } Q>Q_b, \end{array}\right. } \end{aligned}$$

where $\beta U_L = 1.7, \beta U_R =3.2, L_L= 0.15, L_R = 0.08$ and the position of the barrier top is $Q_b = 0.67$.

## Derivation of the asymptotic MFPT formula in equilibrium

Here, we derive Eq. (6) following the methods introduced in [41]. Using the Fourier transform of the GLE in Eq. (1), where we consider a harmonic approximation $U(x) \approx Kx^2/2$, we obtain

B.1 $$\begin{aligned} \tilde{x}(\omega ) = \frac{\tilde{F}_R(\omega )}{K - m \omega ^2 + i \omega \tilde{\Gamma }^+ ( \omega )} \equiv \tilde{\chi }(\omega ) \tilde{F}_R (\omega ), \end{aligned}$$

where $\tilde{\chi }(\omega )$ is the Fourier-transformed response function. We write the autocorrelation function for the position as

B.2 $$\begin{aligned} \begin{aligned} C(t)&\equiv \langle x(t)x(0) \rangle = \int \frac{d \omega }{2 \pi } e^{i \omega t } \int \frac{d \omega ' }{2 \pi } \langle \tilde{x}(\omega ) \tilde{x}(\omega ')\rangle \\&= \int \frac{d \omega }{2 \pi } e^{i \omega t } \int \frac{d \omega '}{2 \pi } \tilde{\chi }(\omega ) \tilde{\chi }(\omega ')\langle \tilde{F}_R(\omega ) \tilde{F}_R(\omega ')\rangle \\&= k_BT \int \frac{d \omega }{2 \pi } e^{i \omega t } \int \frac{d \omega ' }{2 \pi } 2\pi \delta (\omega + \omega ')\tilde{\Gamma }(\omega ) \tilde{\chi }(\omega ) \tilde{\chi }(\omega ')\\&= k_BT \int \frac{d \omega }{2 \pi } e^{i \omega t } \tilde{\Gamma }(\omega ) \tilde{\chi }(\omega ) \tilde{\chi }(-\omega ), \end{aligned} \end{aligned}$$

where we used $\langle \tilde{F}_R(\omega ) \tilde{F}_R(\omega ')\rangle = k_BT 2 \pi \delta (\omega +\omega ') \tilde{\Gamma }(\omega )$. From this, the Fourier transform of the autocorrelation function follows as

B.3 $$\begin{aligned} \tilde{C}(\omega ) = \beta ^{-1} \tilde{\Gamma }(\omega ) \tilde{\chi }(\omega ) \tilde{\chi }(-\omega ) . \end{aligned}$$

The half-sided Fourier transform $\tilde{\Gamma }^{+}(\omega )$ of the memory kernel $\Gamma (t)$ is given by

B.4 $$\begin{aligned} \tilde{\Gamma }^+(\omega ) = \int ^\infty _0 dt e^{-i\omega t} \Gamma (t) = \frac{\gamma }{1 + i \omega \tau }, \end{aligned}$$

and hence

B.5 $$\begin{aligned} \tilde{\Gamma }(\omega ) = \tilde{\Gamma }^+ (\omega ) +\tilde{\Gamma }^+ (-\omega ) = \frac{2 \gamma }{1 + \omega ^2 \tau ^2}. \end{aligned}$$

Inserting $\tilde{\Gamma }(\omega )$ and $\tilde{\chi }(\omega )$ into Eq. (B.3), we obtain

B.6 $$\begin{aligned} \tilde{C}(\omega )= \frac{2 \gamma \beta ^{-1}(1 + \omega ^2\tau ^2)^{-1}}{\left( K - \omega ^2\left[ m - \frac{\tau \gamma }{1 + \tau ^2 \omega ^2}\right] \right) ^2 + \frac{\omega ^2 \gamma ^2}{(1+ \omega ^2\tau ^2)^2}}, \end{aligned}$$

which can be rewritten in a form that corresponds to the standard result for the memoryless (i.e., $\tau =0$) harmonic oscillator in equilibrium as

B.7 $$\begin{aligned} \tilde{C}(\omega ) = \frac{2 \gamma _{\text {eff}} \beta ^{-1}}{(K - m_{\text {eff}} \omega ^2)^2 + \omega ^2 \gamma _{\text {eff}}^2}, \end{aligned}$$

where we defined effective friction and mass as

B.8a $$\begin{aligned} m_{\text {eff}}&= m - c_1\tau \gamma _{\text {eff}} \end{aligned}$$

B.8b $$\begin{aligned} \gamma _{\text {eff}}&= \frac{\gamma }{1+c_2\tau ^2 \omega ^2 } \end{aligned}$$

and where we introduced numerical constants $c_1$ and $c_2$ that account for the harmonic approximation of the potential. In the low-friction limit, Eq. (B.7) is dominated by the pole

B.9 $$\begin{aligned} \omega ^2_{L}= K/m_{\text {eff}} \quad \textrm{for} \quad \frac{K m_{\text {eff}}}{\gamma _{\text {eff}}^2} >1. \end{aligned}$$

Inserting Eqs. (B.9) and (B.8a) into Eq. (B.8b), we arrive at

$$\begin{aligned} \gamma ^L_{\text {eff}} =&\frac{\gamma }{1 + \frac{c_2\tau ^2K}{m - c_1\tau \gamma ^L_{\text {eff}}}},\\ \end{aligned}$$

which corresponds to the quadratic equation

$$\begin{aligned} {\gamma _{\text {eff}}^L}^2 - \gamma _{\text {eff}}^L \left( \frac{m}{c_1\tau } + \frac{c_2}{c_1}\tau K + \gamma \right) + \frac{\gamma m}{c_1\tau } = 0\\ \end{aligned}$$

with solutions

$$\begin{aligned} \gamma _{\text {eff}}^L =&\frac{m}{2c_1\tau } + \frac{c_2\tau K}{2c_1} + \frac{\gamma }{2 }\\&\pm \sqrt{\left( \frac{m}{2c_1 \tau } +\frac{c_2\tau K}{2c_1}-\frac{\gamma }{2}\right) ^2+ \frac{c_2}{c_1}\tau K \gamma }. \end{aligned}$$

In the limit $\tau \rightarrow 0$, we obtain

$$\begin{aligned}&\gamma _{\text {eff}}^L = \frac{m}{2c_1\tau } + \frac{ c_2\tau K}{2c_1} + \frac{\gamma }{2 }\\&\pm \frac{m}{2c_1 \tau }\left[ \left( 1 + c_2\frac{\tau ^2 K}{m} - \frac{c_1\tau \gamma }{m}\right) ^2 + \frac{4 c_1 c_2\tau ^3 K \gamma }{m^2}\right] ^{1/2}\\&\simeq \frac{m}{2c_1\tau } + \frac{c_2 \tau K}{2c_1} + \frac{\gamma }{2 }\\&\pm \frac{m}{2c_1 \tau }\left[ 1 - \frac{2c_1 \tau \gamma }{m } + \frac{2 c_2\tau ^2 K}{m} + \frac{c_1^2\tau ^2 \gamma ^2}{m^2} +\frac{2c_1 c_2 \tau ^3 K \gamma }{m^2}\right] ^{1/2}\\&\simeq \frac{m}{2c_1\tau } + \frac{c_2 \tau K}{2c_1} + \frac{\gamma }{2 }\\&\pm \frac{m}{2c_1 \tau }\left( 1 - \frac{c_1 \tau \gamma }{m } + \frac{ c_2\tau ^2 K}{m} +\frac{2 c_1 c_2\tau ^3 K \gamma }{m^2}\right) . \end{aligned}$$

In order to recover the limiting behavior $\gamma _{\text {eff}}^L=\gamma$ for $\tau \rightarrow 0$, we take the minus sign, which leads to

B.10 $$\begin{aligned} \gamma _{\text {eff}}^L \simeq \gamma - \gamma \frac{c_2\tau ^2 K}{m} . \end{aligned}$$

In the limit $\tau \rightarrow \infty$, we obtain

$$\begin{aligned}&\gamma _{\text {eff}}^L = \frac{m}{2c_1\tau } + \frac{c_2\tau K}{2c_1} + \frac{\gamma }{2 } \\&\pm \frac{c_2\tau K}{2c_1 }\left[ \left( 1 + \frac{c_1 \gamma }{ c_2\tau K} + \frac{m}{ c_2\tau ^2 K}\right) ^2 - \frac{4c_1 \gamma m}{ c_2^2\tau ^3 K^2}\right] ^{1/2}\\&\simeq \frac{m}{2c_1\tau } + \frac{c_2 \tau K}{2c_1} + \frac{\gamma }{2 } \\&\pm \frac{ c_2\tau K}{2c_1 }\left[ 1 + \frac{2c_1\gamma }{c_2\tau K } + \frac{2 m}{c_2\tau ^2 K } + \frac{c_1^2 \gamma ^2}{c_2^2\tau ^2 K^2}\right. \\&\left. + \frac{2c_1 \gamma m }{c_2^2\tau ^3 K^2}- \frac{4 c_1 \gamma m}{c_2^2\tau ^3 K^2}\right] ^{1/2}\\&\simeq \frac{m}{2c_1\tau } + \frac{c_2 \tau K}{2c_1} + \frac{\gamma }{2 } \\&\pm \frac{c_2 \tau K}{2c_1 }\left[ 1 + \frac{2c_1 \gamma }{c_2 \tau K } + \frac{2 m}{c_2 \tau ^2 K } + \frac{c_1^2\gamma ^2}{c_2^2\tau ^2 K^2} - \frac{2c_1 \gamma m }{c_2^2\tau ^3 K^2}\right] ^{1/2}\\&\simeq \frac{m}{2c_1\tau } + \frac{c_2\tau K}{2c_1} + \frac{\gamma }{2 }\\&\pm \frac{c_2\tau K}{2c_1}\left( 1 + \frac{c_1 \gamma }{c_2\tau K } + \frac{ m}{c_2\tau ^2 K } - \frac{2c_1 \gamma m }{c_2^2\tau ^3 K^2}\right) . \end{aligned}$$

Again, in order to recover the limiting behavior $\gamma _{\text {eff}}^L=0$ for $\tau \rightarrow \infty$, we take the minus sign, which leads to

B.11 $$\begin{aligned} \Rightarrow \gamma _{\text {eff}}^L \simeq \frac{\gamma m}{\tau ^2 K} . \end{aligned}$$

Combining the two limits Eqs. (B.10)–(B.11), we arrive at the expression

B.12 $$\begin{aligned} \gamma _{\text {eff}}^L&= \frac{\gamma }{1 + \frac{c_2\tau ^2 K}{m}} \quad \text {and} \end{aligned}$$

B.13 $$\begin{aligned} m^L_{\text {eff}}&= m-c_1\tau \gamma _{\text {eff}}^L. \end{aligned}$$

In the high-friction limit, Eq. (B.7) is dominated by the pole

B.14 $$\begin{aligned} \omega ^2_{H}=- K^2/\gamma ^2_{\text {eff}} \quad \textrm{for} \quad \frac{K m_{\text {eff}}}{\gamma _{\text {eff}}^2} <1. \end{aligned}$$

Inserting this expression into Eq. (B.8b), we obtain

B.15 $$\begin{aligned} \gamma _{\text {eff}}^H&=\frac{\gamma }{1 - c_2\tau ^2 K^2/{\gamma _{\text {eff}}^H}^2}= \frac{\gamma {\gamma _{\text {eff}}^H}^2}{{\gamma _{\text {eff}}^H}^2- c_2\tau ^2 K^2}, \end{aligned}$$

which leads to the following quadratic equation

$$\begin{aligned} {\gamma _{\text {eff}}^H}^2 - \gamma \gamma _{\text {eff}}^H -c_2\tau ^2 K^2 =0 \end{aligned}$$

with solutions

B.16 $$\begin{aligned} \gamma _{\text {eff}}^H&= \frac{\gamma }{2} \pm \sqrt{\frac{\gamma ^2}{4} +c_2\tau ^2 K^2}. \end{aligned}$$

Here, in order to recover the limiting behavior $\gamma _{\text {eff}}^H=\gamma$ for $\tau \rightarrow 0$ we take the plus sign, which leads to

B.17 $$\begin{aligned} \gamma _{\text {eff}}^H&=\frac{\gamma }{2}\left[ 1 + \left( 1+ \frac{4c_2\tau ^2 K^2 }{\gamma ^2}\right) ^{1/2} \right] \simeq \gamma \quad \text {and } \end{aligned}$$

B.18 $$\begin{aligned} m^H_{\text {eff}}&= m-c_1\tau \gamma . \end{aligned}$$

To avoid a negative value in Eq. (B.8a), we combine the negative term in Eq. (B.13) with the high-friction term in Eq. (5) to obtain the speed-up term, according to $\frac{\gamma }{K}-\tau \simeq \frac{\gamma }{K}\left( \frac{1}{1+K \tau /\gamma }\right)$. Fitting the prediction for the MFPT with inserted effective parameters to the simulation data, we determine the optimal values $c_1 =8(\beta U_0)^2/3$ and $c_2 = 2/3$ [41], where the constants $d_1$ and $d_2$ are determined from the asymptotic Kramers limits and are given in the main text [4, 59].

## Comparison of different asymptotic formulas for the MFPT with simulations

In Fig. 8 we compare the asymptotic formula for the well-to-well MFPT Eq. 6 with two slightly different versions presented earlier in literature. For proper comparison, we compare with simulation results for well-to-well transitions in symmetric potentials. For a single-component exponential memory kernel, the formula presented by Kappler et al. [39] is given by

C.1 $$\begin{aligned} \begin{aligned}&\tau _{MFP} = e^{\beta U_0}\left[ \frac{1}{\beta U_0}\left( \tau _m + 4\beta U_0 \frac{\tau ^2}{\tau _D}\right) \right. \\&\quad \quad \quad +\left. \frac{\pi }{2 \sqrt{2}\beta U_0}\frac{\tau _D}{1+ 10 \beta U_0\tau /\tau _D} + \frac{2}{\beta U_0} \sqrt{\beta U_0 \frac{\tau _m}{\tau _D}}\tau _D\right] , \end{aligned}\nonumber \\ \end{aligned}$$

which is shown as dashed lines in Fig. 8. Lavacchi et al. suggested the following form [41]

C.2 $$\begin{aligned} \begin{aligned} \tau _{MFP}&= e^{\beta U_0} \left[ \frac{1}{\beta U_0} \frac{3 \pi }{8\sqrt{2}} \left( \frac{m}{\gamma } + \frac{2K \tau ^2}{3\gamma }\right) \right. \\&\quad +\left. \frac{2\sqrt{2}\pi \gamma }{K}\frac{1}{1+ 2K \tau /(\beta U_0 \gamma )} + 4 \sqrt{2\frac{ m}{K}}\right] , \end{aligned} \nonumber \\ \end{aligned}$$

shown as dashed-dotted lines in Fig. 8. Equation (6) is shown as solid lines. The main difference between the three formulas is revealed in the speed-up regime for low particle mass and large barrier height. From Fig. 8, it is seen that Eq. (6) describes the data slightly better than the other formula. Equation (C.1) shows slight deviations from the simulation data at the minimum for small mass in Fig. 8a), while Eq. (C.2) does not describe the data well for large barrier heights in Fig. 8b).

## Grote-Hynes theory

The Grote–Hynes (GH) theory predicts barrier-crossing times in the presence of frequency-dependent friction as [9]

D1 $$\begin{aligned} \tau ^{GH}_{MFP} = \frac{2 \pi \omega _{\text {max}}}{\lambda \omega _{\text {min}}}e^{\beta U_0}, \end{aligned}$$

where $\omega _{\text {max}}= \sqrt{|U^{\prime \prime }_{\text {max}}|/m}$ and $\omega _{\text {min}} = \sqrt{|U^{\prime \prime }_{\text {min}}|/m}$ are the frequencies at the potential maximum and minimum. In Eq. (D1), we recognize a factor corresponding to the transition-state theory (TST) prediction,

D2 $$\begin{aligned} \tau ^{TST} = \frac{2\pi }{\omega _{\text {min}}}e^{\beta U_0}. \end{aligned}$$

For a symmetric double-well potential given by Eq. (4), i.e., for $U_0=U_L=U_R$, the frequencies are $\omega _{\text {max}} = \sqrt{4\beta U_0/(\tau _D \tau _m)}$ and $\omega _{\text {min}}= \sqrt{8\beta U_0/(\tau _D \tau _m)}$. $\lambda$ is the barrier reactive frequency, which is determined by the GH equation

D3 $$\begin{aligned} \lambda ^2 + \lambda \frac{\tilde{\Gamma }(\lambda )}{m} = \omega ^2_{\text {max}}, \end{aligned}$$

where $\tilde{\Gamma }(\lambda )$ is the Laplace transform of the friction memory kernel. For a single-exponential memory kernel given by Eq. (3), we have

D4 $$\begin{aligned} \tilde{\Gamma }(\lambda ) = \int _0^\infty \Gamma (t^\prime )e^{-\lambda t^\prime } dt^\prime = \frac{\gamma }{1 +\tau \lambda }. \end{aligned}$$

Inserting the Laplace transform of the single-exponential friction memory kernel into the GH equation (D3), we obtain a cubic polynomial, which has a real and positive root.

In Fig. 9, we observe good agreement between GH theory and the simulation data for short rescaled memory time $\tau /\tau _D$. However, the GH prediction deviates from the simulation results for intermediate and long memory times. To better understand this, we study the various limits of GH theory.

The Markov limit ($\tau /\tau _D\rightarrow 0$) is equivalent to the Kramers (Kr) intermediate–high-friction limit. Here $\tilde{\Gamma }(\lambda )=\gamma$, so that Eq. (D4) simplifies to

D5 $$\begin{aligned} \lambda = -\frac{\gamma }{2 m} \pm \left( \frac{\gamma ^2}{4m^2} +\omega ^2_{\text {max}}\right) ^{1/2}. \end{aligned}$$

Taking the positive root, we obtain

D6 $$\begin{aligned} \tau ^{Kr} = \omega _{\text {max}}\left[ \left( \frac{\gamma ^2}{4m^2} +\omega ^2_{\text {max}}\right) ^{1/2}-\frac{\gamma }{2 m}\right] ^{-1} \tau ^{TST}. \end{aligned}$$

In the Markovian high-friction limit $(\frac{\gamma }{m}\gg 1)$, we obtain

D7 $$\begin{aligned} \lambda&= \frac{\gamma }{2 m}\left( 1 + \frac{4 m^2 \omega ^2_{\text {max}}}{\gamma ^2}\right) ^{1/2} - \frac{\gamma }{2 m}\nonumber \\&\simeq \frac{\gamma }{2 m }+ \frac{m \omega ^2_{\text {max}}}{\gamma } - \frac{\gamma }{2 m} = \frac{m \omega ^2_{\text {max}}}{\gamma } \end{aligned}$$

and from that

D8 $$\begin{aligned} \tau ^{Kr}_{\text {hf}} = \frac{2 \pi \gamma }{m \omega _{\text {max}}\omega _{\text {min}}}e^{\beta U_0}. \end{aligned}$$

In the Markovian low-friction limit $(\frac{\gamma }{m}\ll 1)$, we obtain

D9 $$\begin{aligned} \tau ^{Kr}_{\text {lf}} = \tau ^{TST}, \end{aligned}$$

because $$\lambda = \omega _{\text {max}}$$.

In the low-mass $(m\rightarrow 0)$ limit, the GH equation (D3) becomes

D10 $$\begin{aligned}  &   \lambda \frac{\gamma }{(1 + \tau \lambda )m} -\omega ^2_{\text {max}} =0, \end{aligned}$$

D11 $$\begin{aligned}  &   \Rightarrow \lambda = \frac{\omega ^2_\text {max} m/\gamma }{1 - \tau \omega ^2_\text {max} m/\gamma }, \end{aligned}$$

so that we obtain for the barrier-crossing time

D12 $$\begin{aligned} \tau ^{GH}_{\text {low-mass}}=\frac{2\pi (1 - \omega ^2_{max}\tau m/\gamma )}{\omega _{\text {min}}\omega _{\text {max}}m/\gamma }e^{\beta U_0}, \end{aligned}$$

which recovers the speed-up regime for intermediate $\tau$.

In the long-memory-time limit $(\tau /\tau _D\gg 1)$, the Laplace transform of the memory kernel is

D13 $$\begin{aligned} \tilde{\Gamma }(\lambda ) \simeq \frac{\gamma }{\tau \lambda } \end{aligned}$$

so that

D14 $$\begin{aligned} \lambda = \omega _{\text {max}}\left( 1 - \frac{\gamma }{\tau \omega ^2_{\text {max}}}\right) ^{1/2} \end{aligned}$$

and the GH prediction becomes

D15 $$\begin{aligned} \tau ^{GH}_{\text {long memory}} = \frac{2\pi }{\omega _{\text {min}}\left( 1-\frac{\gamma }{\tau m\omega ^2_{\text {max}}}\right) ^{1/2}}e^{\beta U_0}. \end{aligned}$$

In the limit $\tau \rightarrow \infty$, the barrier-crossing time becomes independent of $\tau$ and is given by

D16 $$\begin{aligned} \tau ^{GH}_{\tau \rightarrow \infty }=\frac{2\pi \sqrt{m}}{\sqrt{U^{\prime \prime }_{\text {min}}}}, \end{aligned}$$

which is reflected by the saturation of the GH prediction in Fig. 9. It is observed that in the double limit $\tau \rightarrow \infty$ and $m\rightarrow 0$, the GH barrier-crossing time prediction goes to zero, in contrast to Eq. (6).

The simulation data and Eq. (6) in Fig. 9 exhibit a minimum at a finite memory time. Assuming a large barrier height, $10 \beta U_0\tau /\tau _D\gg 1$, and low mass, $m\rightarrow 0$, Eq. (6) yields

D17 $$\begin{aligned} \begin{aligned} \tau _{MFP}&= e^{\beta U_0} \left[ \frac{1}{\beta U_0} \frac{3 \pi }{8\sqrt{2}} \frac{2K \tau ^2}{3\gamma } +\frac{2\sqrt{2}\pi \gamma }{K}\frac{1}{ K\beta U_0 \tau /(4\gamma )}\right] . \end{aligned} \end{aligned}$$

In this double limit, the memory time at the minimum, determined by $\partial \tau _{MFP} / \partial \tau |_{\tau = \tau ^*}=0$, is given by

D18 $$\begin{aligned} \tau ^* = \frac{\root 3 \of {32}\gamma }{K}, \end{aligned}$$

and thus is finite and goes to zero as $U_0\rightarrow \infty$. The value of the mean first-passage time at the minimum is

D19 $$\begin{aligned} \tau _{MFP}( \tau ^*) = \frac{e^{\beta U_0}}{\beta U_0} \frac{\pi \gamma }{K} \frac{ 3 (\root 3 \of {4})^2}{\sqrt{2}}. \end{aligned}$$

## Barrier-crossing kinetics for non-equilibrium systems

Here we follow recent calculations for the barrier-crossing dynamics of non-equilibrium systems in symmetric double-well potentials [41]. For a non-equilibrium system, the Fourier transform of the position is given by

E1 $$\begin{aligned} \tilde{x}(\omega ) = \frac{\tilde{F}_R(\omega )}{K - m \omega ^2 + i \omega \tilde{\Gamma }^+_V ( \omega )} \equiv \tilde{\chi }(\omega ) \tilde{F}_R (\omega ), \end{aligned}$$

which defines the response function $\tilde{\chi }(\omega )$. The half-sided Fourier transform $\tilde{\Gamma }^{+}_V(\omega )$ of $\Gamma _V(t)$ is given by

E2 $$\begin{aligned} \tilde{\Gamma }^+_V (\omega ) = \int ^\infty _0 dt e^{-i\omega t} \Gamma _V(t) = \frac{\gamma }{1 + i \omega \tau _V}, \end{aligned}$$

while the Fourier transform of the symmetric random-force correlation $\Gamma _R(t)$ is

E3 $$\begin{aligned} \tilde{\Gamma }_R (\omega ) = \tilde{\Gamma }_R^+ (\omega ) +\tilde{\Gamma }_R^+ (-\omega ) = \frac{2 \gamma }{1 + \omega ^2 \tau _R^2}. \end{aligned}$$

The Fourier transform of *C*(*t*) is given by $\tilde{C}(\omega ) = \beta ^{-1} \tilde{\Gamma }_R(\omega ) \tilde{\chi }(\omega ) \tilde{\chi }(-\omega )$ and reads

E4 $$\begin{aligned} \tilde{C}(\omega )= \frac{2 \gamma \beta ^{-1}(1 + \omega ^2\tau _R^2)^{-1}}{\left( K - \omega ^2\left[ m - \frac{\tau _V \gamma }{1 + \tau _V^2 \omega ^2}\right] \right) ^2 + \frac{\omega ^2 \gamma ^2}{(1+ \omega ^2\tau _V^2)^2}}. \end{aligned}$$

We introduce effective parameters to rewrite Eq. (E4) in the form of the standard memory-less harmonic oscillator in equilibrium [41]

E5 $$\begin{aligned} \tilde{C}(\omega ) = \frac{2 \gamma _{\text {eff}} \beta _{\text {eff}}^{-1}}{(K - m_{\text {eff}} \omega ^2)^2 + \omega ^2 \gamma _{\text {eff}}^2}, \end{aligned}$$

where we have introduced effective frequency-dependent friction, mass, and temperature as

E6a $$\begin{aligned} m_{\text {eff}} = m - c_1\tau _V \gamma _{\text {eff}}, \end{aligned}$$

E6b $$\begin{aligned} \gamma _{\text {eff}} = \frac{\gamma }{1+c_2 \tau _V^2 \omega ^2 }, \end{aligned}$$

E6c $$\begin{aligned} \beta _{\text {eff}} = \frac{1 + \omega ^2 \tau _R^2}{1 + \omega ^2 \tau _V^2}\beta \end{aligned}$$

and where we introduced numerical constants $c_1$ and $c_2$ that account for the harmonic approximation of the potential, as in Appendix B. For the effective mass and the friction in the high- and low-friction limits, we obtain the same results as in Appendix B, where the memory time $\tau$ is replaced by $\tau _V$. For the effective temperature, we insert Eq. (B.9) and Eq. (B.14) into Eq. (E6c) and obtain in the low- and high-friction limits

E7 $$\begin{aligned}&\frac{\beta _\textrm{eff}^L}{\beta } = \frac{\tau _R^2+ m/K -c_1\tau _V \gamma _\textrm{eff}^L/ K }{\tau _V^2 + m/K -c_1\tau _V \gamma _\textrm{eff}^L/ K }, \end{aligned}$$

E8 $$\begin{aligned}&\frac{\beta _\textrm{eff}^H}{\beta } = \frac{\tau _R^2 - (\gamma _\textrm{eff}^H/ K)^2 }{\tau _V^2 - (\gamma _\textrm{eff}^H /K)^2 }. \end{aligned}$$

In the limits $\tau _m/\tau _V <1$, $\tau _m/\tau _R <1$, $\gamma /(K \tau _V) <1$, $\gamma /(K \tau _R) <1$, which hold for the simulated systems, we obtain

E9 $$\begin{aligned} \beta _{\text {NEQ}}/\beta = \tau _R^2/\tau _V^2 \approx \beta _\textrm{eff}^L/\beta \approx \beta _\textrm{eff}^H/\beta . \end{aligned}$$
